# An Enhanced Backbone-Assisted Reliable Framework for Wireless Sensor Networks

**DOI:** 10.3390/s100301619

**Published:** 2010-03-01

**Authors:** Ali Tufail, Syed Ali Khayam, Muhammad Taqi Raza, Amna Ali, Ki-Hyung Kim

**Affiliations:** 1 Graduate School of Information and Communication, Ajou University, Suwon, Korea; E-Mails: ali_tufail@ajou.ac.kr (A.T.); amna_ali@ajou.ac.kr (A.A.); 2 School of Electrical Engineering and Computer Science, NUST, Islamabad, Pakistan; E-Mail: ali.kayam@seecs.edu.pk; 3 USN Networking Research Team, Electronics and Telecommunications Research Institute (ETRI), Korea; E-Mail: taqi@etri.re.kr

**Keywords:** Wireless Sensor Network (WSN), sensor nodes, reliability, backbone routers

## Abstract

An extremely reliable source to sink communication is required for most of the contemporary WSN applications especially pertaining to military, healthcare and disaster-recovery. However, due to their intrinsic energy, bandwidth and computational constraints, Wireless Sensor Networks (WSNs) encounter several challenges in reliable source to sink communication. In this paper, we present a novel reliable topology that uses reliable hotlines between sensor gateways to boost the reliability of end-to-end transmissions. This reliable and efficient routing alternative reduces the number of average hops from source to the sink. We prove, with the help of analytical evaluation, that communication using hotlines is considerably more reliable than traditional WSN routing. We use reliability theory to analyze the cost and benefit of adding gateway nodes to a backbone-assisted WSN. However, in hotline assisted routing some scenarios where source and the sink are just a couple of hops away might bring more latency, therefore, we present a Signature Based Routing (SBR) scheme. SBR enables the gateways to make intelligent routing decisions, based upon the derived signature, hence providing lesser end-to-end delay between source to the sink communication. Finally, we evaluate our proposed hotline based topology with the help of a simulation tool and show that the proposed topology provides manifold increase in end-to-end reliability.

## 1. Introduction

Recent developments in Wireless Sensor Networks (WSNs) have brought this domain from merely a concept of microelectronics to a new realm of practical applications. A typical WSN comprises of a large number of low powered, low cost, memory/computationally-constrained, intelligent sensor devices. These sensors are generally involved in detecting and measuring some target phenomena.

Due to its inherent energy, footprint and deployment constraints, a WSN is prone to faults and malfunctioning. These faults can be due to hardware/software failures or energy depletion. In hostile deployments, the faults may be caused by natural or human adversaries, e.g., natural disasters in calamity-struck regions or radio jamming in a battlefield [1]. Despite WSN’s fault-prone characteristics, mission-critical natures of emerging WSN applications (e.g., military, healthcare, and disaster recovery applications) require that communication to/from sensors is dependable and reliable. The source to sink communication in WSNs is generally dependent on the intermediate relaying sensor nodes. Therefore the reliability of a transmission is dependent on the topology and routing techniques being deployed in the WSN.

Generally, applications require either packet driven reliability (like intrusion detection applications, battlefield surveillance applications etc) or event driven reliability (like habitat monitoring applications, temperature recording applications etc). Packet driven reliability demands that all the packets must be delivered successfully from the source to the sink, whereas event driven reliability requires the event to be detected even with the help of one packet delivery. In this paper, we focus on the requirement of packet driven reliability applications. We propose to enhance WSN routing reliability using high-reliability hotline links between sensor gateways. A WSN typically contains multiple resourceful gateway nodes that provide load balancing, local cluster management and energy saving [2–4]. Since these gateways are far fewer in number than the sensor nodes, we introduce the concept of using hotlines (e.g., high speed Ethernet links) for inter-gateway communication, while traditional multi-hop techniques can be used by the sensors for intra-gateway communication. With multiple hotline-connected gateways we are able to achieve reliable source to sink communication. This paper describes this proposed topology in detail. This is followed by analytical modeling and comparison of traditional WSN communications *versus* hotline-based approach. Our analytical results show that significant improvements in reliability can be achieved using a hotline based topology.

In this paper we also introduce a Signature Based Routing (SBR) scheme, that vests intelligence to those resourceful gateway nodes. SBR is presented as a remedy to delay problems that arise in our suggested hotline assisted routing approach [5]. SBR gives an alternative routing path for all those scenarios where source and the sink are just couple of hops away. In other words, if the source to sink communication is better, in terms of end-to-end latency, via all wireless path then SBR uses that route otherwise the hotline path is used. Gateways keep the track of the latency of all the communication between the source and the sink and derive a threshold (Signature). This signature then helps to improve end-to-end latency for the subsequent communication between the source and the sink. Signature value is recomputed after every source to sink communication. Our simulation results suggest that SBR scheme helps to reduce the overall end-to-end delay.

The rest of this paper is organized as follows. Section 2 outlines related work in this area. Section 3 describes our network model and assumptions and explains hotline-based reliable communication topology. Section 4 focuses on the mathematical evaluation and comparison of the proposed topology with existing WSN communications. Section 5 gives an overview of the Reliability Theory and discusses the effect of cost with regard to achieved reliability. Section 6 introduces the SBR scheme. Section 7 shows an overview of the simulation results. Section 8 summarizes key conclusions of this work and our future directions.

## 2. Related Work

### 2.1. Multiple Gateways for WSNs

The nodes deployed in a WSN are generally large in numbers and are deployed in close proximity. Therefore there is an intrinsic manageability issue in WSNs. Large number of nodes in WSN can be managed by deploying more than one gateway. Multiple gateway based architecture for IPv6-based Low-power Wireless Personal Area Network (6LoWPAN) has been proposed in [6]. Authors in [6] show that their proposed architecture allows a sensor network to achieve better communication performance. Increase in number of gateways would cost additional hardware resources therefore a tradeoff is required. In [7], the authors propose an intelligent estimation approach to calculate least number of gateways required to fulfill certain data latency threshold. They also discuss the impact of location of gateways on the operation of the network. With the known least number of gateways, arises the need to position gateways in an appropriate layout to meet certain network parameters. An optimum layout for the position of gateways has been proposed in [8]. Authors show that the location of gateways has a marked influence on the data rate and overall power efficiency of the network.

### 2.2. Load Balancing and Clustering Schemes for WSNs

In [4], an algorithm to divide network sensor nodes into well-defined clusters is proposed. A similar topology was also proposed in [3], where the problem is referred to as the Load-Balanced Clustering Problem (LBCP). They argue that clustering improves the stability and enhances the inter node communication. In [9], authors present a life-time aware load balancing routing protocol. They argue that by embedding the load balancing technique at the network layer provides significant improvements in the lifetime of WSNs.

### 2.3. Reliable and Energy Efficient Routing

The main idea in [10] is of a forwarding scheme for reliable and energy-efficient data delivery in a cluster-based sensor network. [11] presents a delay aware reliable transport (DART) protocol. Authors focus on timely event detection with a focus on congestion and energy efficiency. Reference [12] discusses the tradeoffs of enhancing the reliability of WSN in a ZigBee network. In [13], the concept of multiple gateways for efficient routing and data delivery within the 6lowPAN is proposed. Quite recently an effort has been made to enhance the reliability of WSN using emergency routing paths [14]. Authors in [14] presents an AODV based routing protocol that uses a multipath algorithm to increase the reliability in a sensor network. However, their routing protocol fails to provide reliable packet delivery for a network having high degree of failed or sleeping nodes. Authors in [15] try to improve the reliability and availability of WSNs by proposing a data forwarding algorithm. However, they just focus on low duty cycle WSNs and they have not presented any good comparison of their approach with other existing reliable routing approaches for WSNs.

### 2.4. Small World Network

A wireless network that uses few wired links as opposed to all wireless links is known as small world network, derived from the idea of small world graphs. This network topology has been proposed in [16], where the authors discuss the concept of using wires in some of their links to enhance energy efficiency but they do not evaluate the impact of such a topology on WSN reliability.

### 2.5. Backbone Approaches for WSNs

Limited energy at each node has been widely recognized as the main barrier to the deployment of WSNs. A backbone approach has therefore been considered as a viable option for enhancing the overall lifetime of a WSN. Authors in [17] suggest that a construction of energy efficient backbone prolongs network lifetime, brings stability and scalability. Whereas in [18] authors present Sensor DMAC which reduces the overhead of node selection, backbone formation and maintenance, thus increases the overall network lifetime. [19] talks about a protocol for building and maintaining a connected backbone. Their design criteria produce a backbone that can be reconfigured quickly with very little overhead. In [20], authors present a tree construction algorithm that is used to form a stable backbone using multi hop clusters in a WSN. Authors argue that their approach helps to balance the load and energy consumption of a WSN.

### 2.6. Reliability in WSNs

While reliability of homogeneous WSNs has been investigated in prior studies [21–24], reliability analysis of backbone approach in WSN is largely unexplored. In [22], authors formulate a WSN reliability measure that considers the aggregate flow of sensor data into a sink node. This measure is based on a given estimation of the data generation rate and the failure probability of each sensor. Common cause failures (CCF) have been discussed and identified as the cause of unreliability in WSN in [23,24]. Authors in [23] consider the problem of modeling and evaluating the coverage-oriented reliability of WSN subject to CCF whereas in [24] the main emphasis is on addressing the problem of modeling and evaluating the infrastructure communication reliability of WSN. In [21], the authors compute a measure for the expected and maximum message delay between data sources and data sinks in an operational distributed sensor network (DSN). Authors in [25] present a reliable routing protocol that forms the reliable routing path by utilizing network topology and routing information of the network. However, their protocol and analysis is application specific. Moreover, they have not provided any comparison with existing reliability schemes. In [5] we introduced the concept of hotlines to improve the reliability for WSNs. We proved analytically that concept of hotlines enhances the reliability of WSNs manifold. However, we did not provide any simulation experimentations to prove the result. Moreover, as explained previously in Section 1, few scenarios in hotline approach might give more end-to-end latency especially if source and the sink are just few hops away. All these scenarios are not accounted for in [5].

Our work is quite different from the prior work described above. While we have utilized few concepts like clustering and multiple gateways from [4–13], small world graphs from [16], backbone approach from [17–19], and gateway association mechanism from [6], our emphasis is on addressing the reliability issues in WSN using the concept of hotlines between gateways. Most of the previous work, especially in backbone approach; focuses on energy efficiency in WSNs whereas we have combined versatile concepts–like backbone, clustering, hotlines–to simultaneously enhance both reliability and energy efficiency in a WSN. We also propose protocol modifications to achieve the hotline assisted topology and provide a reliability theoretic analysis of the proposed topology. Moreover, our paper suggests a brand new concept of SBR for WSNs to support end-to-end delay requirement of different applications.

## 3. Hotline-Based Reliable WSN Topology

We assume a WSN with two-level heterogeneity. At the first level, we have resource-constrained sensor nodes which are deployed densely on a two-dimensional grid. All of the first level nodes have the same resources. At the second level, we have sensor gateways that operate as cluster heads to regulate the flow of traffic and to manage sensor motes deployed in the given geographical region. The gateways are not resource-constrained and their density is many orders of magnitude lesser than the density of the sensor nodes. Gateways are connected to each other in a bus topology. In the traditional topology, the bus is constructed using long-haul wireless links. Under the proposed hotline-based topology, the gateways are connected via highly reliable links, e.g., Ethernet cables or point-to-point wireless links. We assume a fixed network topology where both gateway nodes and the sensor nodes are static.

We now introduce the important components of our proposed hotline-based reliable WSN topology. Different mechanisms, like dividing the network into clusters, gateway association/dissociation and node-node, node-gateway and gateway-gateway communication will be described in detail in the following sub-sections. The role of multiple gateways can be made useful in WSNs if they are connected by a bus. Gateways are therefore being used to provide an alternative and reliable path for packet routing.

In traditional WSN routing the expected path of end-to-end communication would include multiple hops. Packet delivery is dependent on several intermediate relaying sensor nodes. These sensor nodes forward the packets until they reach their destination. WSNs have inherently high link error rates. With multi-hop routing, the cumulative probability of error increases exponentially with the increasing number of hops [26]. Thus the chances of failure are likely to increase with every additional hop. Reliability is therefore a main concern for mission-critical WSN deployments. Our suggested topology addresses the reliability issue by using the concept of hotlines between gateways. Hotlines reduce the number of average hops between a source and destination and provide better reliability as compared to traditional WSN routing.

**Figure 1.**
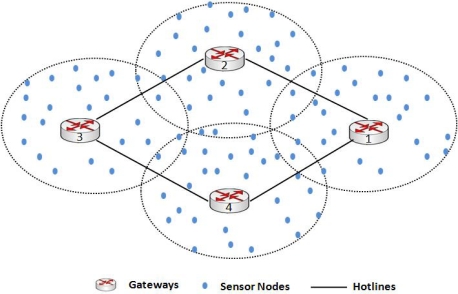
Hotline deployed WSN Topology.

Figure 1 shows an example of our hotline assisted topology where four gateways are connected to each other as a bus network. This bus network is not only connecting gateways to each other but also connecting gateways to outer world (Internet or IPv6 domain). We would describe the topology in detail in the following sections.

### 3.1. Network Clusters

Due to the availability of gateways, we can efficiently organize and manage the sensor nodes in a network. Each node would have to associate to a particular gateway making it the default gateway. All the nodes associated to one gateway form one cluster. Please note that gateway and cluster head are same name used interchangeably.

One of the important benefits of the clustering approach is to facilitate efficient and reliable communication using gateway hotlines. For example, one set of nodes is sensing the environment to get the data and then that data is being sent to another set of nodes or a sink for further computation. The default gateways can provide a fast and reliable routing mechanism to communicate this data.

**Figure 2.**
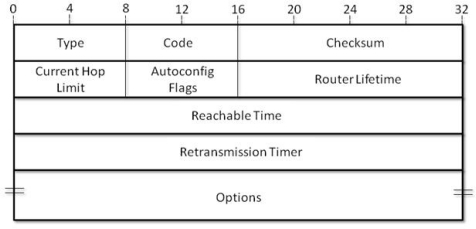
Router Advertisement Message Format [27].

Nodes become the part of a cluster depending upon their hop count from the gateway. The nodes would get associated to any gateway by using the information in the Router Advertisement (RA) message [6]. The routers would send router advertisement (RA) messages to help the nodes to associate to a particular gateway. When a sender node receives RA messages from several routers, it would differentiate the messages with help of current time to live (Cur TTL) field. It must be noted that Cur TTL has been modified. Every router sets the value of Cur TTL field to be 255 and with each hop the value is decreased by 1. So the nodes get associated to the router with a maximum Cur TTL, since a higher value of Cur TTL implies less hop count to that router. The RA message format is shown in the following Figure 2. Our field of interest here is Current Hop Limit Field which is a byte long.

### 3.2. Inter-Node Communication

**Figure 3.**
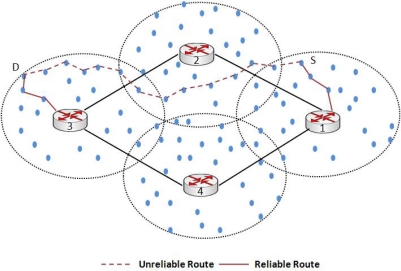
Traditional unreliable route and hotline assisted route.

Under traditional *ad hoc* routing algorithms like AODV and DSR [28], when a node S has to send data to a destination D, routes are discovered using a flooding mechanism. As shown in Figure 3, sender S does not know the path to destination D so the source floods the network with a route request (RREQ). RREQ is then rebroadcasted by every node that receives the message, until it reaches the destination or an intermediate node that has a fresh route to the destination in its cache. The destination node or the intermediate node then replies with a route reply (RREP). As shown in Figure 3, the route with dotted line is one of the routes discovered using a traditional routing algorithm. Due to the large number of intermediate wireless hops, a message sent through that path would likely be dropped due to wireless channel errors and will also be incurring high end-to-end delay.

In our proposed topology, as we have divided the network into different clusters and each cluster is associated with a default gateway, routing of packets and route discovery is done within the cluster using RREQs. Our approach of intra-cluster routing is closer to the approach suggested in [13]. (We recommend OSPF for gateway-to-gateway routing, as will be explained in section 3.4) All the traffic (inter/intra-cluster and to/from the Internet) is routed through the gateway. The only exception here is the border nodes which are discussed later. Instead of an end-to-end wireless path, a packet is now routed through wireless-wired-wireless path, where the wired path is the communication between gateways via hotline and the wireless paths are used for intra-cluster communication. This approach clearly enhances the reliability of an end-to-end transmission. Under traditional routing, the path discovery process will discover an end-to-end wireless path. This path is less reliable, as shown in Figure 3, because the packet will traverse many wireless hops and with every hop there is an inherent threat of packet loss due to a variety of reasons like channel errors, collisions, and dead or sleeping nodes [4]. Moreover, under this approach, a number of sensor nodes are acting as relaying nodes. Consequently, more energy is consumed on sensors which results in a shortened network lifetime.

Figure 3 also shows the hotline-assisted path that provides more reliable routing and involves lesser number of hops than traditional routing. A path discovery (RREQ) message will now reach from sender S to the default gateway. The default gateway will route the RREQ packet to the destination gateway. The destination gateway will then route the packet to the destination. The resultant route is more reliable and helps to conserve the energy of individual nodes thereby increasing the network lifetime.

### 3.3. Border Nodes

*Definition 1: Border nodes* are the nodes that lie on the overlapping region of two or more clusters.

The nodes that lie in the communication range of one or more gateways can associate with either of the gateways depending on different factors (*i.e.,* signal strength, shortest path etc [6]). Moreover, in a scenario where the source to sink communication is only a few hops but source-gateway-gateway-sink communication has more number of hops then it would be expensive to communicate using the longer path (*i.e.,* hotline assisted). It would be expensive in terms of overall latency and energy consumption. This case is likely to be true if both the source and the sink are on the border (or overlapping region) of two different clusters. We call these nodes as border nodes and propose a slightly different routing and gateway association mechanism between them. This mechanism would not only help towards less latency between the source and the sink but also would provide an efficient gateway association. The first task is therefore to identify the border nodes.

**Figure 4.**
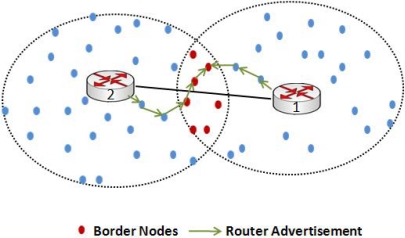
Identifying Border nodes.

For simplicity, we take the example of two clusters and two associated default gateways. It can be seen from Figure 4 that nodes in the overlapping region of two clusters are defined as border nodes. The sample node lies in the overlapping region of cluster 1 and cluster 2 because it receives two RA messages from two routers. This node is now responsible for two decisions one is to join a particular gateway and the other is to broadcast or forward the RA messages of the gateways. This decision depends on the value of Cur TTL field in the RA message. Let us take an example scenario. If the node gets a RA message from gateway 1 with Cur TTL value of 252 and another RA message from gateway 2 with Cur TTL of 250, the node would join the gateway with a higher value of Cur TTL and at the same time would broadcast the RA message. Note that the Cur TTL field represents the hop count towards the gateway.

In a scenario where a node receives two RA messages from two different gateways with the same value of Cur TTL it can join either of the gateways. The node would forward both the RA messages to its neighboring nodes.

If border nodes have to send a packet, for instance, they would check from the neighbor table whether the packet is destined to their first hop neighbor. If the packet is destined to the first hop neighbor they would deliver it directly no matter the neighbor belongs to other cluster. This way the latency would reduce and for the applications requiring prompt response, this communication would be more reliable. The comparison of routes is shown in the figure below. However, this approach is focused only on the special case of the border nodes. In order to cover the complete network for the problem of increased end-to-end latency, we present a generic routing scheme SBR in section 6.

### 3.4. Inter-Gateway Communication

The gateways are connected using high-speed Ethernet cables. Gateways are supposed to exchange certain information that would be required for routing and monitoring the state of the links. Our sensor network has been divided into clusters but overall network can be considered as one autonomous system (AS). We therefore require a protocol configured in gateways that can handle cluster to cluster (Intra-AS) communication. We propose to employ the widely-used Open Shortest Path First (OSPF) protocol for inter-gateway communication. We choose OSPF because of two main reasons: 1) In distance vector routing protocols, each router does not possess information about the complete network topology and consequently there is a slow convergence problem [29]; 2) OSPF works over IP and has a richer set of extensions and added features [29] as compared to other link state and distance vector routing protocols.

Each gateway has information saved about nodes of the complete network in form of a gateway network table. Every time a node is added or removed from a particular cluster, an update is sent to the default gateway. The default gateway accordingly updates the gateway network table. An update message is eventually sent to all the gateways of the network connected via the hotline. This completes one cycle of the update mechanism. This process is already supported in OSPF by the database synchronization mechanism [29]. The synchronization process begins as soon as the gateway attempts to bring up the adjacency. The database is described by each gateway by sending a series of Database Description packets to its neighbors. As gateways are connected in a bus network, via hotline, all the gateways will synchronize their databases.

Gateways are also deployed with a monitoring mechanism. Specifically, gateways are aware of the health of neighboring gateways and the corresponding links. This monitoring mechanism helps to ensure the connectivity of the gateways and eventually ensuring the availability of the hotline. This mechanism is supported by the OSPF Hello protocol. The neighbor relationship is maintained with the help of the Hello Protocol. It is also responsible of ensuring the bidirectional communication between neighbors. Hello packets are sent periodically on all router interfaces [29].

In summary, gateways are configured with dual functionality. They are deployed with two different protocols. One protocol (AODV) is used for intra-cluster or cluster-node communication, the other protocol (OSPF) is used for inter-gateway communication.

## 4. Reliability Model and Evaluation

In this section, we model and compare the reliability of an end-to-end WSN communication under traditional and hotline-assisted routing.

Our network has two different homogenous Poisson distributions. The normal sensor nodes are deployed according to a dense homogeneous Poisson distribution and gateway nodes are deployed according to a sparse homogenous Poisson distribution. Throughout this section, we consider a one-dimensional deployment scenario for simplicity.

**Figure 6.**
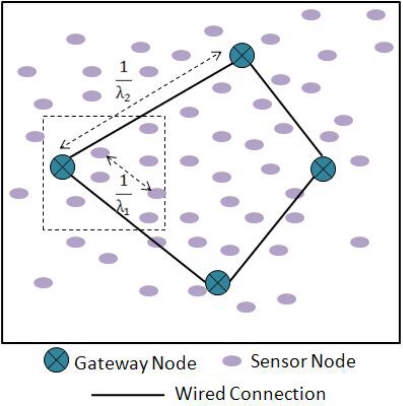
Network model showing distribution scenario of sensor nodes and sensor gateway nodes.

We assume that there are λ_1_ arrivals of sensor nodes per unit area. In other words there are, on the average, λ_1_ occurrences of sensor nodes per unit area. As shown in Figure 6, we can conclude that the average distance between two neighboring nodes in a unit distance will be 1λ1. Similarly on the average there are λ_2_ occurrences of sensor gateway nodes per unit distance. The average distance between two neighboring sensor gateway node is therefore 1λ2. The gateway nodes are connected to each other via a backbone wired link and the occurrences of gateway nodes per unit distance are significantly lesser than that of sensor nodes, leading to the following relation: λ_2_ ≪ λ_1_.

Alternatively, we can write the above equation as:

(1) $$\lambda_{2}=\alpha \lambda_{1}$$

where 0 < *α* <1 is the ratio of the gateway node occurrence with respect to the number of sensor nodes per unit distance. The value of *α* would vary depending upon specific scenario or the density of sensor nodes and sensor gateway nodes in a given network. If *α* is closer to 1 then the network is expected to be a well managed network with a high number of gateway nodes.

**Figure 7.**
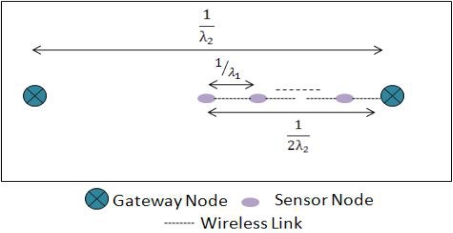
Worst case scenario with a sensor node lying exactly in the middle of two gateway nodes.

We want to compute the reliability and resilience of our proposed topology against the traditional WSN routing mechanism. We take the worst case scenario where we consider a border node that lies exactly at the center of two gateway nodes as shown in Figure 7. As we already know that the average distance between two gateway nodes is 1λ2. The node that lies right in the middle of two gateway nodes would have 12λ2 distance to either of the gateway nodes. But we know that the average distance between two neighboring sensor nodes is 1λ1. Using (1), we obtain: 12λ2=12α1λ1. In the above equation 12α is the factor that provides us required number of 1λ1’s to cover the whole distance of 12λ2.

**Figure 8.**
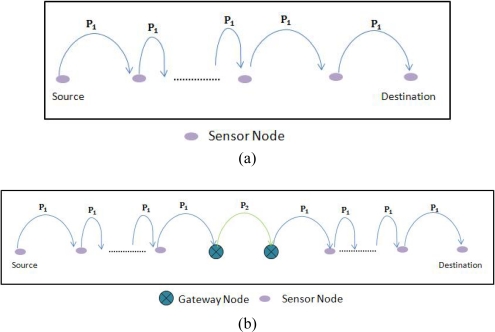
(a) Probability of reliable packet transmission without gateway nodes. (b) Probability of reliable packet transmission with gateway nodes.

We are now interested in quantifying the reliability of the traditional and hotline-assisted scenarios. The reliability of communication between a sender and a receiver is the end-to-end probability of successful transmission. As shown in Figure 8(a), there is no gateway available so the reliability would depend on the number of hops, *n*, between the sender and the destination. Each of the hops would have the same probability of successful packet transmission; we call this probability *P*_1_. The reliability without gateway nodes is then: (*P*_1_)*^n^*

In order to find out the reliability of the scenario when gateway nodes are assisting the routing, the overall reliability would be dependent on three different phases. One is the probability of packet reaching from source to the associated source gateway, say *P*_1_, second is the probability of packet reaching from the source gateway to the destination gateway, say *P*_2_, and lastly the probability of packet reaching from the associated destination gateway to the destination, which in worst-case is *P*_1_. This scenario is shown in the Figure 8(b).

Please note that in order to simplify the evaluation and comparison we have assumed that the probability of any packet traversing through wireless media (no matter from sensor node to sensor node or sensor node to gateway node) is P_1_ and that of the wired media is P_2_ we have not considered some factors like sleep state, transmission power, queue length, buffer overflow etc.

We know that in our particular example the probability of successful transmission of packets on wireless domain, where they also have more number of hops, would be much less than that of the hotline-assisted gateway-to-gateway transmission. So we now have the relation: *P*_1_ ≪ *P*_2_. The above equation can be written as: *P*_1_ = *βP*_2_, where 0 < *β* < 1.

The probability of successful transmission in hotline assisted scenario is:

(2) (P1)12α(P2)h(P1)12α=(P1)1α(P2)h

where *h* is the number of wired hops between source and destination gateway nodes. The minimum value of *h* is 1.

In case of traditional all wireless routing, the distance covered by the wired path in a hotline-assisted routing scenario will be replaced by a wireless path. We already know that the average distance between two neighboring gateway nodes is 1λ2 as shown in the Figure 4. From equation (3) we know that 1α represent the number of wireless hops in the given scenario. To cover the whole distance of *h* wired hops, we need the following number of wireless hops:

Average Number of Replaced Hops=h×1α

In order to get probability for all wireless successful packet transmission we would get the following equation:

(3) (P1)12a(P2)ha(P1)12a=(P1)(h+1)a

If we compare Equation (2) and (3) we would get:

(P1)1a(P2)h≠(P1)(h+1)a(P2)h≠(P1)ha.

If we evaluate the above equation we can find out the reliability of both the scenarios as highlighted in Figure 8(a) and (b). Since *P*_2_ ≫ *P*_1_, 0 < *α* < 1, and of *h* is a positive integer, we have

(4) (P2)h≫(P1)ha

From Equation (2) and (3) we can assume that the hotline assisted reliability is represented by **R**_h_ and traditional routing reliability is represented by **R***_t_*. From Equation (4) we know that the following relation should hold: **R**_h_ ≫ **R**_t_.

**Figure 9.**
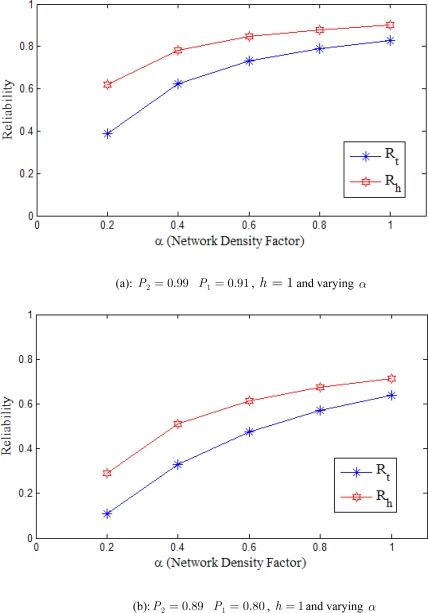

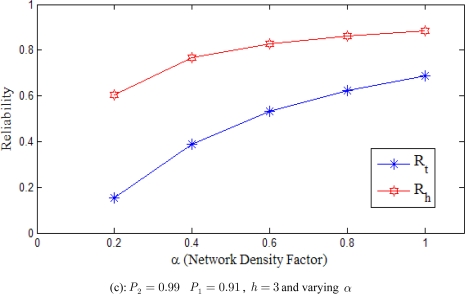
Comparison of hotline based reliability with traditional wireless reliability.

Figure 9 compares the reliability of packet transmission in the case when routing is supported by our proposed hotline assisted gateways with the case when the routing is done in a traditional manner. The graphs are drawn against varying values of *α*.

In Figure 9(a) and (b), we use *h* = 1. In other words, the number of wired hops between the source and destination gateways is one. Figure 9(a) shows that in case of hotline assisted routing, where the probability of successful packet transmission is assumed to be 0.99, the reliability (**R**_h_) would be dependent on the value of *α*; or the relative densities of sensor and gateway nodes. An increase in *α* would increase the reliability of WSN and vice versa. If the value of *α* is less, automatically the deployed gateway density reduces, and therefore the reliability of WSN is low. Although in the case of traditional all wireless routing, where the probability of successful packet transmission is assumed to be 0.91, the reliability (**R***_t_*) is dependent on the value of *α*, but **R***_t_* is always much less than **R**_h_ with any given value of *α*. This is due to the fact that hotline assisted topology is routing packets through more reliable (wired) links and fewer un-reliable (wireless) hops, Traditional routing, on the other hand, is routing packets through end-to-end unreliable wireless hops.

In Figure 9(b), we have kept all the factors constant but have reduced the probability of successful packet transmission for both *P*_1_ and *P*_2_. The graph shows that the value of **R**_h_ is still high than the value of **R***_t_* for any given value of *α*. Please note that the reliability is much less than that provided in 9(a) due to the fact that reliability in our proposed approach is directly proportional to the probability of successful packet transmission, provided value of *h* is constant. With very low value of *α* the value of **R***_t_* is observed to be very low and is much lesser than that of **R**_h_, therefore traditional routing provides much less reliability as compared to the proposed hotline assisted gateway routing.

In the Figure 9(c) we have increased the number of wired hops (*i.e., h*) from 1 to 3. The increase in wired hops has triggered a dramatic decrease in the overall reliability of traditional all wireless routing (**R***_t_*). This is a direct consequence of the fact that in the traditional (all wireless) scenario many new wireless hops are needed to cover the distance that would be covered by the hotlines in our proposed topology. These new wireless hops are inherently unreliable and result in significantly increased packet losses. A slight decrease in the reliability of hotline assisted routing is also observed in Figure 9(c). This reduction in reliability is, nevertheless, negligible in comparison with the reliability penalties incurred by the all-wireless traditional routing scheme.

## 5. Evaluation of Reliability Cost

In this section, we first overview the reliability theory and then later in the section we try to evaluate the cost of achieving reliability for a WSN.

### 5.1. Background on Reliability Theory

The reliability theory provides a model of a multi-component system that can be used to find the probability that the system is functional under a given set of constraints. The theory models the system as a set of components connected in series, parallel or hybrid (series-parallel) configurations. The reliability of the complete system is then computed as a function of the reliability of the individual components. In this section, we introduce basic concepts and definitions used in reliability theory literature [30].

#### 5.1.1. Structure functions

Let indicator variable *x_i_* be the state of *i^th^* component. Mathematically,

$$x_{i}=\{\begin{array}{ll} 1, & \text{if}\;i^{\mathrm{th}}\;\text{component is functional}\;\\ 0, & \text{if}\;i^{\mathrm{th}}\;\text{component has failed} \end{array}$$

A state vector *X* = {*x*_1_, *x*_2_, . . ., *x_n_*} shows indicator variables of all *n* components of a system. From the state vector, one can determine whether the overall system is functional or failed.

A *structure function ϕ*(*X*) then determines the working of a system by utilizing *X*. Mathematically, *ϕ*(*X*) is defined as:

$$\phi (X)=\{\begin{array}{ll} 1, & \text{if the system is functional when the}\\ & \text{state vector is}\;X\\ 0, & \text{if the system has failed when the}\\ & \text{state vector is}\;X \end{array}$$

#### 5.1.2. System and component reliabilities

The state of *i^th^* component can also be represented by a random variable *X_i_* such that *P*{*X_i_* = 1} = *p_i_* = 1 − *P*{*X_i_* = 0}, where *p_i_* is the probability that *i^th^* component is functional, also referred as its *reliability*. The overall reliability of a system is then

$$r\;=\;P\phi (X)\;=\;1\},\;\mathrm{where}\;X\;=\;X_{1},X_{2},\;.\;.\;.\;,\;X_{n}\}\;.$$

If *p* = {*p*_1_, *p*_2_, *p*_3_, . . ., *p_n_*} is a state vector representing the reliabilities of individual components, the system’s reliability function is *r* = *r*(*p*).

#### 5.1.3. Series and parallel systems

**Figure 10.**
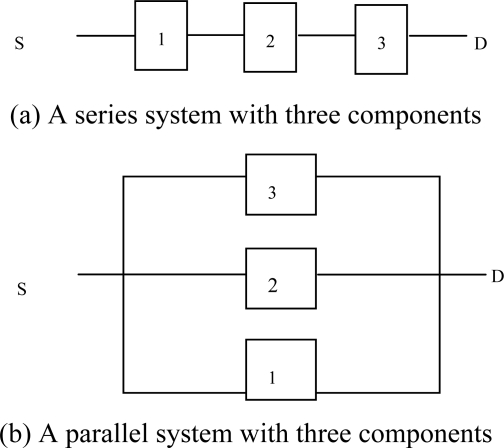
A series and a parallel system.

A series system consists of *n* components connected in a series fashion as shown in Figure 10(a). Such a system is only functional if all of its components are functional. The reliability function *r*(*p*) of a series system is

(5) $$r(p)\;=\;\prod_{i=1}^{n}p_{i}.$$

A parallel system, on the other hand, has components connected in a parallel fashion (Figure 10 (b)). In this case, the system is functional if at least one of its components is functional. Therefore, the reliability function of a parallel system is

(6) $$r(p)\;=1-\;\prod_{i=1}^{n}\left(1-p_{i}\right).$$

#### 5.1.4. Definitions

*Definition 1: Backbone to sensor ratio* is the ratio of backbone nodes to wireless nodes in a given unit area of a WSN.
*Definition 2:* A *subsystem* is a path between a <source, destination> pair in a WSN.
*Definition 3:* Sensor nodes in a WSN are the *routing components* or simply the *components* of the WSN subsystem.
*Definition 4:* Backbone nodes in a WSN are the *backbone routing component* of the WSN subsystem.
*Definition 5:* Reliability of a WSN is the probability of successful transmission of packets between a <source, destination> pair.

### 5.2. Reliability Cost

We propose the use of backbone routing for WSN to improve the overall reliability of the network. The backbone is formed at the cost of additional gateway nodes. Since each additional component in the system is generally treated as an overhead in resource-constrained WSN, we define the *cost* of a system as:

*Definition 6: Cost* of a system is the total number of components in the system.

A network is said to be *connected* if every pair of nodes is connected through at least one path. Our system model assumes that the network is connected through a subsystem of *t* − 1 components. We further assume that subsystem contains a finite number of components and hence its reliability is well above 0.

A packet sent by node *S* must traverse at least all *t* − 1 components of a subsystem before being delivered to node *D*. Therefore, the reliability of a subsystem *r_s_*(*p*) is given by

(7) $$r_{s}(p,t)=p_{f}^{t-1}.$$

To study the reliability of a system, we assume that *m* subsystems exist between a <source, destination> pair. Since the subsystems are independent of each other, the system is functional (*r*(*p*) = *P*{ϕ(*X*) = 1}) if all the components of a subsystem are functional; *i.e. r*(*p*) = *P*{*X_i_* = 1 *for some i* =1, . . ., *m*}, where *X_i_* is the indicator variable of a subsystem. Mathematically, *r*(*p*) is equivalent to 1 − (*P*{*X_i_* = 1 *for all i* =1, . . ., *m*}) Hence, using (6) and (7), the reliability of the system *r_P_*(*m*, *p_f_*, *t*) is

(8) $$r_{P}(m,p_{f},t)=1-{\left(1-p_{f}^{t}\right)}^{m}.$$

The cost of the system is therefore given by

(9) C(m,t)=m(t−1).

**Figure 11.**
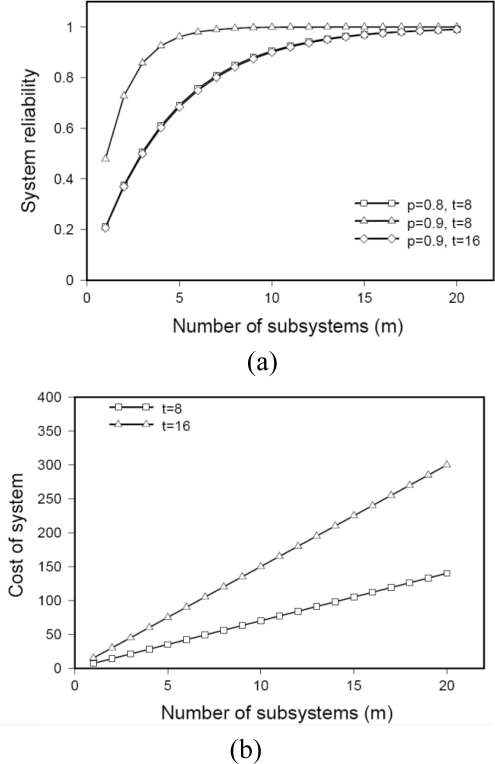
A comparison of the system reliability and the associated cost. (a) Reliability of the system as a function of the number subsystems. (b) Cost of the system as a function of the number subsystems.

We plot (8) against different values of *m* to observe the increase in overall system reliability along with its associated cost (9). The results of functions (*r_P_*(*m*, *p_f_*, *t*)) and *C*(*m*, *t*)) for varying values of *p_f_*, *t* and *m* are plotted in Figure 10. Note that the reliability of the system increases exponentially and then saturates after a certain number of additional subsystems. Thus the reliability of a system does not improve if we add more subsystems. While reliability benefits are negligible, cost (see Figure 11(b)) of the system keeps increasing linearly with the addition of more subsystems. This would be more obvious if the components being added are resource rich and expensive like gateway nodes.

## 6. Signature Based Routing

In hotline assisted routing all the packets from sender to the receiver are routed through the gateways [5]. This path may sometimes increase the end-to-end latency. The hotlines therefore provide the reliability on the cost of increased latency in certain cases. We have observed the following case where there might be chances of increased end-to-end latency:

1. In cases where the destination node is just few hops away from the sender node and the communication takes place through hotline. The packet is sent from the sender (probably through multi hops) to its default gateway and then via the destination’s default gateway the packet reaches the destination (probably again via multi hops). Therefore the hotline path might bring more end-to-end latency in few cases.

In the following section we present the remedy for above mentioned latency issue.

In order to avoid longer source to sink paths and increased latency we present Signature Based Routing (SBR) scheme. It is a known fact that general sensor nodes are mainly resource constrained devices. Putting those devices into performing additional computation (say calculating additional or optimal routing paths) would not be a good idea. On the other hand we require shorter and intelligent but reliable routing from source to the sink. As pointed out earlier, there are few cases where source to the sink direct communication is less costly as compared to the hotline path; we therefore present intelligent routing where gateways play a central role. We call this routing signature based, as the routing decision is made by the gateway depending upon comparison results with specific signatures.

*Definition 7: Personal Area Network (PAN)* is defined in accordance to IEEE 802.15.4 standard. PAN comprises of sets of low power nodes and a PAN coordinator. In our case resource rich gateways act as the PAN coordinators.

In our hotline based approach we have suggested all the communication from source to the sink goes via gateways. It means all the traffic to and from a PAN goes through gateways. Moreover gateways are usually resource rich devices as compared to general sensor nodes [31]. As explained earlier, gateways share the complete network topology information in the form of gateway network tables. Therefore gateways can easily monitor the traffic, find out the source-to-sink latency of each packet and can take routing decisions based upon certain criterion [31].

**Figure 12.**
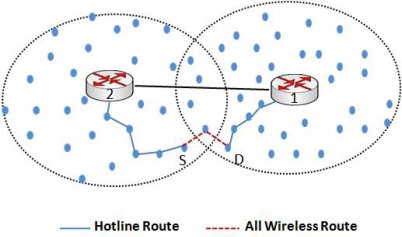
Scenario where the direct communication is better as compared to hotline routing.

In order to explain SBR we take the following example scenario. As shown in Figure 12 the source and the sink happen to be close enough that if they communicate directly, instead of communicating via hotline, it will not only decrease the latency but also will increase the reliability as it reduces number of wireless hops it would have required to reach from sender to the gateway and gateway to the sink. However, there are several other cases where source to sink communication is much more reliable with less latency, if the communication goes through the hotlines. Therefore, we vest intelligence in gateways to monitor traffic and take appropriate routing decisions (*i.e.,* to decide if the routing is better via hotlines or directly via all wireless path).

In above scenario the source needs to send the packet to the destination (*i.e.,* sink), initially it follows the default route, source-gateway-gateway-destination. However, both the involved gateways keep the track of the latency of the each communication between the source and the sink. Gateways compare the latency with the signature value (we will explain signature value later in the section), if the latency is less than the signature value then the communication continue un-interrupted. However, if the latency of X consecutive packets stays more than the signature value then the gateways force the sender and the destination to follow their suggested path. (Here we assume that the gateways are fully aware of the network topology, node position and have the ability to calculate shortest path between the sender and the destination). The value of X can be fixed depending upon the requirements of the WSN or the application. Few applications require least possible latency so the value of X will be equal to 1, whereas some other applications are able to bear more latency so they will set the value of X equal to 2,3,4…. and so forth.

Signature is defined as follows:

(10) $$S=\frac{\sum_{i=1}^{n}\varphi_{i}}{n}$$

where *S* represents the signature, *i* represents the number of gateways in a WSN and *φ* represents the weighted average latency of each PAN calculated by individual gateways for their respective PANs. Each gateway keeps track of the end-to-end latency of every packet that goes through it, so *φ* is the average of all those latencies. To get an idea of overall latency of the complete network we take average of the sum of *φ* of all the PANs. It constitutes our signature. We can calculate the new value of *φ*:

(11) $$\varphi_{j}=\frac{w_{i}\times \varphi_{i}+x_{j}}{w_{i}+1}$$

where *x_j_* represents the current latency observation, *φ_j_* represents the new signature value, *φ_i_* represents the old signature value, and *w_i_* represents the associated weight, the value of *w_i_* is the number of previously recorded latency values excluding the current observation. Please note that weight helps to calculate a stable signature value.

**Figure 13.**
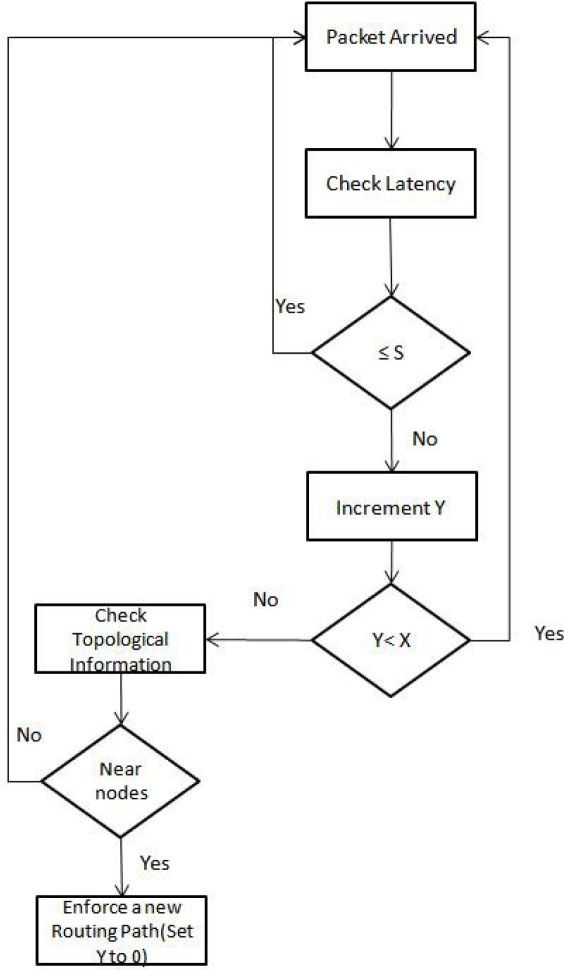
SBR process taking place at gateways. *S* is the signature, *X* represents the threshold value and *Y* represents the current packet count.

In order to understand the flow of information and decision process we can refer to Figure 13. It shows the flow after the packet arrives at the gateway. As shown in Figure 13, before enforcing the new routing path gateways first check topological information. The decision box “near nodes” means that gateways decide if the source and the destination nodes, under consideration, are close enough, in terms of number of hops. Let’s say, there is a source to sink communication scenario where observed end-to-end latency stays more than the signature value for *X* consecutive times. As explained earlier, this should trigger the new path enforcement process. Let’s say the source and destination are on opposite ends of the two PANs. It means all wireless path, probable new path, might have more number of hops. This new path is expected to have more end to end latency as compared to original path. Therefore, gateways decide not to enforce the new path. On the contrary, if the source and the destination are close enough in terms of number of hops, they are expected to have less latency so gateways would enforce the new path. This phase is shown in figure 13 by the decision box “Near nodes”.

**Figure 14.**
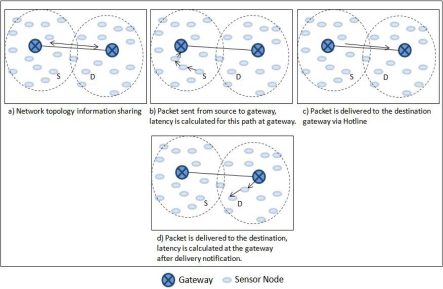
Stepwise overview of the SBR scheme (Phase 1: Hotline assisted Routing).

**Figure 15.**
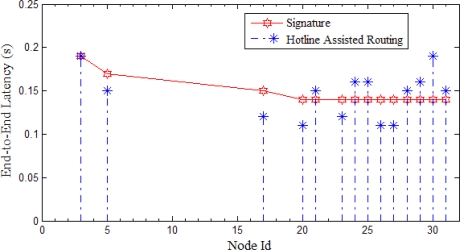
Simulation results showing the end-to-end latency in the case packets are sent using hotline assisted routing. Signature value is also shown.

We now explain the SBR process in detail. We follow an example scenario. The process is divided into two phases for explanation purpose. The first phase involves the hotline assisted routing. The second phase deals with the newly enforced signature based routing. Latency is measured and compared for both the cases. A simulation is preformed for both the cases and will be discussed separately with each phase (Please note that the detailed evaluation of SBR scheme will be discussed later in the evaluation section of the paper). All the nodes in a PAN send the packets to a fixed destination node of the other PAN. However, for the discussion purpose we follow one source to sink communication, so in Figure 14, S represents that source and D represents that destination. Figure 15 shows the corresponding simulation results showing all the sources sending packets to the destination, where the node that we are following is node 3 (*i.e.,* node S).

Figure 14a shows the topology information sharing phase where the gateways share their node information with each other. Please note that *φ* value for every PAN is updated every time a communication takes place through their respective gateway. This update process takes place at the stage shown in Figure 14b and 14d. However, the *φ* is not updated if the latency is more than the signature value. This action keeps the signature either equal or less than the predefined (or application required) latency level (latency level is represented by the value of signature). The value of *φ* is also updated after the communication takes place through the new routing path (*i.e.,* path forced by the gateways). However, signature *S* is recomputed only after the *φ* values are shared among gateways. This sharing can be periodic or after every *φ* update. The update of *φ* and re-computation of *S* makes sure that the recent latency is added to the signature and makes the signature adaptive.

If we analyze Figure 15, we notice that the initial signature value is 0.19. We have taken initial signature to the worst latency value shown in our experiments. The signature value is adaptive and is adjusted with every signature re-computation. We can notice that the node 3 has the latency equal to the signature value; at this stage gateways start monitoring the communication between the sender and the receiver. We will later see in the phase II that how gateways decide to enforce the new path. We can find few other nodes having value more than the signature; gateways monitor all those nodes and keep track of the latency. Please note that the signature is being continuously computed with every latency value.

**Figure 16.**
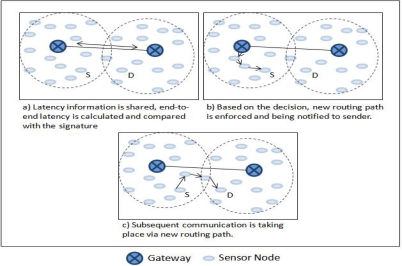
Stepwise overview of the SBR scheme (Phase II: New Path Enforcement).

**Figure 17.**
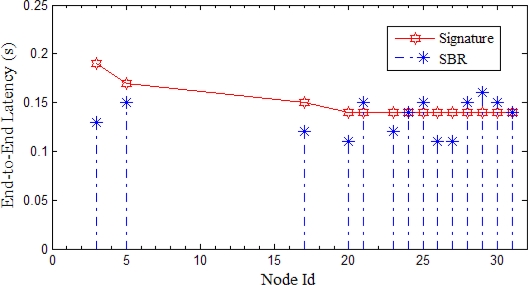
Simulation results showing the end-to-end latency in the case packets are sent using SBR. Signature value is also being shown.

We now review phase II. This phase deals with the process after the gateways start monitoring the nodes with latency greater or equal to the signature. As we saw node 3 had latency greater than signature; it gives the gateway right to decide if it wants to enforce the new SBR path. In order to do that gateway first needs to compare the node positions and see if the nodes are close enough (*i.e.,* in terms of number of hops or distance). If nodes are close then the gateway enforces a new communication path for node 3 (as shown in Figure 16c). Similar procedure will be activated for all those nodes having latency greater than or equal to signature. However, in our simulation node 30 was the only other node that was enforced with a new path (after considering the topological information). Figure 17 shows the results of the simulation after implementing SBR. We can notice that the new path for node 3 (even for node 31) gives a better and more reliable alternative where latency is much less than the previous routing path.

We can conclude that the SBR provides a good addition to our hotline approach [5] where we not only have reliable communication from source to the sink but also with less latency.

## 7. Simulation and Results

In this section we discuss the simulations and their results. Table 1 explains simulation setup and the environment that we utilized to conduct the simulations.

Different kind of routing schemes that we refer in this section are as:

*Definition 8: All wireless routing* refers to the traditional WSN routing scheme that includes only the wireless media to communicate between source to the sink.
*Definition 9: Hotline assisted routing* refers to the reliable routing approach that we suggested where the source to the sink communication is assisted by the high speed hotline links between the gateways.
*Definition 10: Signature Based Routing (SBR)* is the enhanced hotline based routing that reduces the overall latency between the source to the sink communication.
*Definition 11: Multipath AODV Signal Intensity Metric (maodv-sim)[14]* is the routing scheme used for the comparison with our results.

In hotline assisted simulation scenarios the nodes are deployed in clusters as explained in Section 3. The cluster head act as a gateway and all the communication to and from the cluster takes place through the gateway. All the gateways are connected via Ethernet cable. Multiple sources send data to a sink to emulate bursty traffic.

In the all-wireless scenarios we have kept the same network topology and node deployment; however there were no gateways or Ethernet connections.

The factors that are used to measure the reliability of a WSN are 1) Packet Success Ratio and 2) end-to-end Delay.

*Definition 12*: The *Packet Success Ratio* is defined as the ratio between the total number of packets sent by the sender to the total number of packets successfully received by the destination/sink.
*Definition 13: end-to-end delay* is defined as the time taken for a packet to reach from the source to the destination/sink.

If the packets are being dropped frequently, that network will be considered unreliable. In some mission critical applications like military and healthcare immediate communication is required, therefore end-to-end Delay is also considered to be one of the important criterions for measuring the reliability.

We have divided the simulation discussion into three parts. Part A discusses the effect on reliability with respect to varying number of hops from source to the sink, Part B talks about the effect on reliability with respect to varying node density in a specific area and in Part C we compare SBR with all wireless routing and hotline assisted routing in terms of Average end-to-end delay.

### 7.1. Increasing Number of Hops

**Figure 18.**
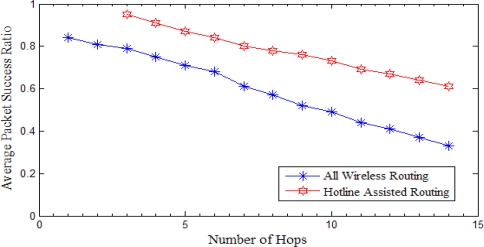
Packet success ratio *versus* number of hops. Hotline assisted routing has fewer hops from source-to-sink due to routing through gateways.

**Figure 19.**
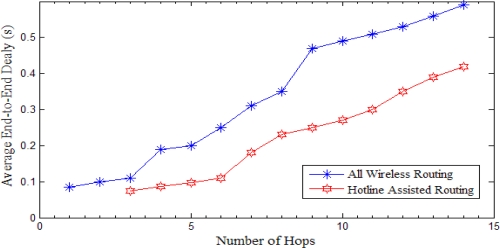
Average end-to-end delay *versus* number of hops. Hotline assisted routing has fewer hops from source-to-sink due to routing through gateways.

Following results are compared with the number of hops between the source and the sink. The results shown in Figure 18 and Figure 19 are the cumulative results of the simulations with regard to the number of hops between the source and the sink.

Please note that the number of hops for hotline assisted routing are different than that of all wireless routing. For example three hops of all wireless routing contain all wireless hops whereas three hops of hotline assisted routing contain at least one wired hop (gateway to gateway). This gateway to gateway wired hop can cover more than one hops of all wireless routing.

#### 7.1.1. Packet Success Ratio

As shown in Figure 18, hotline assisted routing shows much more reliable results compared to all wireless routing. We can notice that as the number of hops is increasing there is a decrease in the packet success ratio in both the cases. However, packet drop rate is much higher in the case of all wireless routing. One of the main reasons is that WSN nodes, following their duty cycle, go to periodic sleep state for energy savings and therefore for multi-hop communications there are more chances that nodes sleeping schedule will clash. We minimize the packet drop in the case of hotline assisted routing with the help of gateway to gateway wired communication. Hotlines bypass the erroneous wireless links and make every gateway just one hop away from all other gateways. Therefore, the proposed topology enhances the packet success ratio manifolds as compared to traditional all wireless routing as shown in Figure 18.

#### 7.1.2. Average End to End Delay

We have shown average end-to-end delay against the number of hops between the source and the sink. As shown in Figure 19 Average end-to-end delay is much less in the case of hotline assisted routing as compared to all wireless routing. We get a huge advantage in terms of less end-to-end delay in the case of hotline assisted routing due to gateway-to-gateway communication (except for few cases as covered by SBR). As the number of hops increases, the delay increases as well. Hotlines avoid these overheads and reduce the overall end-to-end delay as compared to the all wireless routing. These simulation results are very encouraging and pave the way for the deployment of the proposed topology for mission-critical applications encountered in healthcare and military.

### 7.2. Varying Density of the Network

This section focuses on the discussion of simulation results with varying density of nodes. We started the simulation with a sparse network and then noticed the difference in reliability parameters with an increasing node density.

#### 7.2.1. Packet Success Ratio

**Figure 20.**
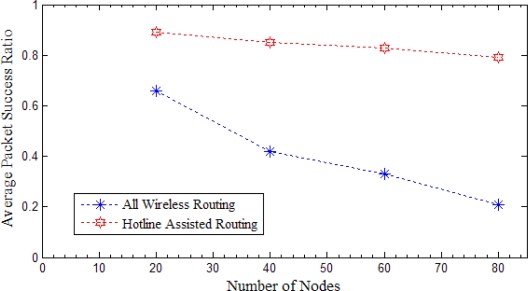
Average Packet Success ratio *versus* number of Nodes

Figure 20 below shows that the hotline assisted routing performs much better as compared to the traditional all wireless routing. As the number of nodes is increasing the packet success ratio in the all wireless routing is decreasing. This is because of the fact that with the increase in density of the network there will be more congestion and interference hence chances of packet loss will be higher [31,32]. On the other hand, we notice that packet success ratio for the hotline assisted routing remains almost unchanged regardless of the network density. The reason behind this is the permanent wired link between any given two gateways. Therefore it will be fair to conclude that communication between the source and the sink is independent of the density of the network in the case of hotline assisted routing.

#### 7.2.2. Average End-to-End Delay

**Figure 21.**
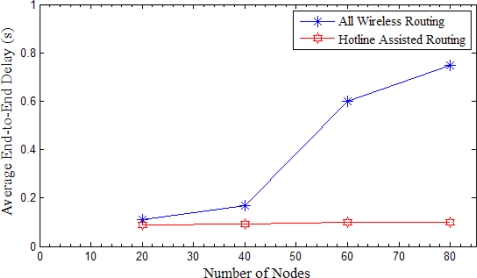
Average End-to-End Delay *versus* number of Nodes.

Figure 21 shows average end-to-end delay *versus* the network density. It is clear even from this figure that hotline assisted routing achieves higher performance as compared to the all wireless routing. Average end-to-end delay stays much lower and almost unchanged with the increasing number of nodes as compared to all wireless routing. The average end-to-end delay increases with the increase in number of nodes in the case of all wireless routing. The probable cause of this raise is the increase in the density of the network. Due to this increase the communication takes longer as compared to a less dense network [32].

### 7.3. Signature Based Routing

**Figure 22.**
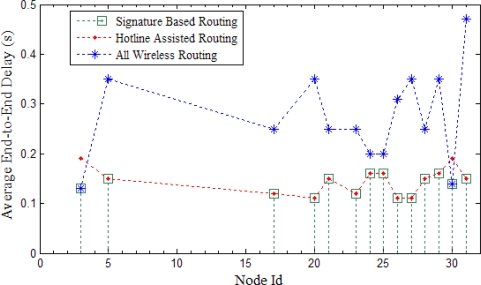
Comparison of SBR with hotline assisted routing and all wireless routing.

In this section, we compare the Signature Based Routing with the hotline assisted routing and all wireless routing. The simulation setup is same as described earlier. Multiple sources send data to a sink. However, for simplicity, in Figure 22 we show the sources of a single PAN. In Figure 22, we show the node Id (please note that the node ids are the same as in our experiment, only the nodes of one PAN are shown here) against Average end-to-end Delay. Each source sent 100 packets to the sink. As we have already explained, SBR rectifies the latency problem for hotline assisted routing. This makes communication reliable with less number of hops. As shown in Figure 22, the value of SBR is either close to all wireless routing or hotline assisted routing. In other words, we can say that for every communication SBR chooses the best of either all wireless routing or hotline assisted routing.

We can see from Figure 22 that SBR is almost the same as hotline assisted routing for all cases except two (*i.e.,* node 3 and node 30). For these two exceptions, SBR adopted the all wireless path as this path has fewer hops. Fewer hops mean more reliability so SBR helps to not only decrease end-to-end delay but also to increase the reliability.

From these simulation results, we conclude that the proposed hotline assisted approach provides much better reliability as compared to the all wireless approach. Therefore, hotline assisted routing can serve as an effective and viable transmission alternative for mission critical applications. Moreover, SBR solves the delay problems of hotline approach making it more robust and reliable.

### 7.4. Performance Comparison

We compare our approach with maodv-sim [14]. Authors in [14] present a routing protocol that enhances the reliability of WSNs by using the technique to find out reliable emergency paths from source to the destination. They have modified the underlying AODV routing protocol to enhance the reliability. To perform an accurate comparison, we have benchmarked the simulation environment as defined in [14]. A total of 50 nodes were deployed in a terrain of 500m×500m. We have used standard MAC Protocol of IEEE 802.15.4, *i.e.* CSMA/CA, as underlying MAC protocol. AODV is used as a routing protocol. The comparison is performed on Average end-to-end latency and Average packet success ratio. We benchmark their best results of reliability achieved by the maodv-sim routing protocol [14]. Node failure is simulated by shutting down the nodes randomly. We compare our suggested SBR with the maodv-sim routing.

**Figure 23.**
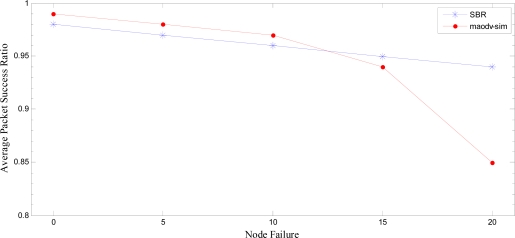
Average Packet Success Ratio comparison of SBR with maodv-sim [14].

Figure 23 shows the average packet delivery ratio plotted against the percentage of failing nodes in the entire network. We notice that with less failed nodes, SBR gives almost the same ratio of packet delivery as claimed by [14]. However, as percentage of node failure increases our scheme outperforms the maodv-sim. Please note that in case of maodv-sim there is a sharp decline in the packet delivery ratio after the node failure rate reaches 15%. On the other hand, the packet delivery ratio in SBR approach is rather stable. Even though maodv-sim uses emergency paths to enhance the reliability but as more nodes start to fail the performance declines many fold. This decline in maodv-sim performance is due to the fact that there is a high probability that emergency path would fail with failing of the next hop nodes and the chances of reaching the destination would be grim. In our scheme the routing from source to the sink includes more reliable gateway assisted routing paths. It makes routing less dependent on the intermediate sensor nodes. Hence, the probability that the failure of sensor nodes will affect hotline based routing is much less in comparison to the routing technique explained in [14]. We notice that at 20% node failure hotline based approach gives almost 10% better packet success ratio as compared to maodv-sim.

**Figure 24.**
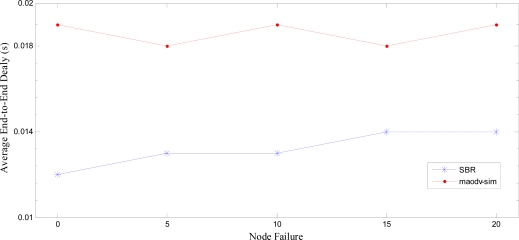
Average End-to-End Delay comparison of SBR with maodv-sim [14]

Figure 24 shows the average end-to-end latency. The overall latency is much less in SBR as compared to maodv-sim [14]. As SBR involves more reliable hotline assisted path, from gateway to gateway, therefore the average end-to-end latency decrease as compared to the traditional routing approach adopted by maodv-sim. Authors in [14] claim that, in case of node failures, the use of pre-determined emergency paths decreases the overall end-to-end latency, nevertheless, performance of SBR outperforms the maodv-sim routing approach as shown in figure 24.

## 8. Conclusion

This paper discusses the issue of reliability in WSNs. We deliberate that the gateway nodes in WSNs can play an important role to improve the reliability of source to sink communications. We proposed a hotline-based topology to enhance the reliability of inter-gateway paths. The proposed topology improved reliability of end-to-end WSN communications by exploiting a concept similar to small-world graphs. We showed through mathematical analysis that hotline assisted routing gives noticeably better reliability in comparison to traditional all-wireless ad hoc routing. Our proposed topology provides reliability which is independent of the number of nodes in a given cluster or area. This property makes it a good choice for high density WSN deployments. We have also observed the impact of additional cost with respect to the achieved reliability. Our SBR scheme provides lesser delays from source to the sink communication. SBR vests intelligence to the gateways and help them make smart routing decisions. This property makes our topology ideal for all the data critical applications. Finally we simulated the topology and showed that our proposed topology achieves a marked improvement in the reliability of WSN in comparison to all wireless routing. Our simulation results show at least 50% improvement in the reliability as compared to the all wireless routing.

## Figures and Tables

**Figure 5.**
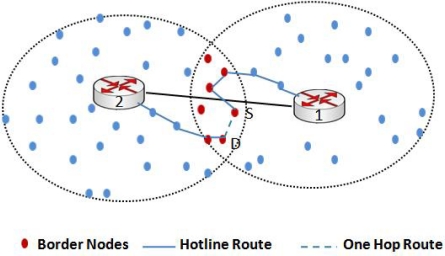
Border nodes can communicate to each other directly.

**Table 1.** Simulation setup.

Simulation Environment	Qualnet 4.5
Routing Protocol	AODV*
Inter-Gateway Routing	OSPF
Intra-Cluster Communication	Wireless (802.15.4)
Inter-Cluster Communication	Wired (Ethernet)
Number of Nodes	20, 40, 60, 80
Total Terrain Area	1500m × 1500m
Simulation Time	60 seconds
Total Runs	20

* In order to make AODV work efficiently in WSN environment we slightly modified its route discovery mechanism in our simulation. For example route discovery area has been partitioned and influx of extra control messages into the network has been monitored and controlled.
